# Assessment of formats and completeness of paper-based referral letters among urban hospitals in Rwanda: a retrospective baseline study

**DOI:** 10.1186/s12913-022-08845-y

**Published:** 2022-11-28

**Authors:** Zamzam Kalume, Bart Jansen, Marc Nyssen, Jan Cornelis, Frank Verbeke, Jean Paul Niyoyita

**Affiliations:** 1 Department of Electronics and Informatics (ETRO), Vrije Universiteit Brussel (VUB), Pleinlaan 2, Brussel, 1050 Belgium; 2 Department of Health Informatics, College of Medicine and Health Sciences, University of Rwanda, PO Box: 3286, Kigali, Rwanda

**Keywords:** Patient referral, Referral letters, Referral completeness

## Abstract

**Background:**

Patient referral is a process in which a healthcare provider decides to seek assistance due to the limitations of available skills, resources and services offered locally. Paper-based referrals predominantly used in low-income countries hardly follow any procedure. This causes a major gap in communication, coordination, and continuity of care between primary and specialized levels, leading to poor access, delay, duplication and unnecessary costs. The goal of this study is to assess the formats and completeness of existing paper-based referral letters in order to improve health information exchange, coordination, and continuity of care.

**Methods:**

A retrospective exploratory research was conducted in eight public and three private healthcare facilities in the city of Kigali from May to October 2021. A purposive sampling method was used to select hospitals and referral letters from patients’ files. A data capture sheet was designed according to the contents of the referral letters and the resulting responses were analyzed descriptively.

**Results:**

In public hospitals, five types of updated referral letters were available, in total agreement with World Health Organization (WHO) standards of which two (neonatal transfer form and patient monitoring transfer form) were not used. There was also one old format that was used by most hospitals and another format designed and used by a district hospital (DH) separately. Three formats were designed and used by private hospitals (PH) individually. A total of 2,304 referral letters were perused and the results show that “external transfer” forms were completed at 58.8%; “antenatal, delivery, and postnatal external transfer” forms at 47.5%; “internal transfer” forms at 46.6%; “Referral/counter referral” forms at 46.0%; district hospital referrals (DH2) at 73.4%. Referrals by private hospitals (PH1, PH2 and PH3) were completed at 97.7%, 70.7%, and 0.0% respectively. The major completeness deficit was observed in counter referral information for all hospitals.

**Conclusion:**

We observed inconsistencies in the format of the available referral letters used by public hospitals, moreover some of them were incompatible with WHO standards. Additionally, there were deficits in the completeness of all types of paper-based referral letters in use. There is a need for standardization and to disseminate the national patient referral guideline in public hospitals with emphasis on referral feedback, referral registry, triage, archiving and a need for regular training in all organizations.

**Supplementary Information:**

The online version contains supplementary material available at 10.1186/s12913-022-08845-y.

## Background

Organizing a healthcare system and optimally providing care are intricate undertakings whereby every single decision is critical. Preferably, every patient should receive quality healthcare services as delineated by the World Health Organization (WHO): *“the right care, at the right time, responding to the service users’ needs and preferences, while minimizing harm and resource waste*” [1]. Primary care has a central role and responsibility for the patient care planning, most importantly, to record critical clinical information and decide when to refer a patient to another health professional. These transfers of care require a complex approach and well-elaborated processes with clear ownership and responsibility of each party [2]. This is an important concept of care coordination whereby healthcare professionals work together to ensure patients receive all the services needed, completed by the continuity of care in which comprehensible and well-organized health care events are performed across a variety of health settings [3]. Hence, patient information exchange is vital to achieve quality service delivery through appropriate coordination and continuity of care [4, 5].

Rwanda is a landlocked country situated in the east of Africa. In the north, Rwanda is bordered by Uganda, in the east by Tanzania, the south by Burundi and the west by the Democratic Republic of Congo. The health care system structure in Rwanda is composed of a primary health care level with 45,516 community health workers, 476 health posts and 499 health centers; a secondary health care level with 36 district hospitals; and a tertiary health care level with 4 provincial hospitals and 8 referral hospitals. The complexity of care increases from lower to higher levels whereby in the primary care level, community health workers provide prevention and promotion of curative health services; health posts provide immunization, family planning, growth monitoring and antenatal care services; health centers provide preventive primary health care, in-patient care, referrals, and basic maternity services. At the secondary care level, district hospitals provide in- and outpatient services, surgery, laboratory services, gynecology-obstetrics, radiology, mental health, dental and eye services. Finally, at the tertiary level, provincial and referral hospitals offer state of the art specialized care. In addition to the above-mentioned public health system, there are 250 registered private health facilities providing different levels of care distributed over dispensaries, clinics, and polyclinics [6]. Due to the limitations of skills, resources and services offered at the lower levels, the referral system is fundamental towards effective and efficient coordination and continuity of healthcare service delivery. Currently, the referral system is paper based and the patients themselves are the carriers of the referral letters. The initial patient record resides at the primary care level. A referral feedback should be received to complete the referral cycle. A community health worker (CHW) can refer to a health post or health center whichever is the nearest health facility for the patient. At the same time a patient can directly visit a health post or health center without a referral letter from the CHW. Health posts refer to the health centers located in the same sector and furthermore, health centers refer to the district hospitals located in the same district. Finally, the district hospitals may refer to any provincial hospital or referral hospital. Private clinics and polyclinics refer to district hospitals, provincial hospitals, or referral hospitals. With exception of emergency cases, a patient needs a referral letter to benefit from medical care of health facilities of superior category [7].

Patient referral is a clinical action based on a decision whereby a health care provider hands over, transfers, or refers a patient to another health care provider in the same or different health settings for the patient to obtain an appropriate diagnosis or treatment [8]. One major reason for deciding to refer is the limited set of services offered at lower levels. Therefore, initiating referral to seek expert opinions, more developed diagnostic investigations and special therapeutic services is paramount for a patient to access advanced care. The mechanisms of the referral system that function at different levels are based on national laws and policies and rely on efficient lines of communication by creating clear, simple steps and procedures for the harmonization of unique standardized protocols with follow-up guidelines [8, 9]. According to the Rwandan community-based health insurance (CBHI), every patient should visit the primary care and if it is considered necessary, get referred to the secondary level and only the secondary care can refer to the tertiary level [7].

Referral letters are acknowledged as a means of health information exchange or communication between primary care physicians and specialists. Different studies argued about qualified referral letters which depend generally on the content of information [10–12]. The quantity and quality of information contained in the referral letters have a considerable impact on the coordination and continuity of care and hence also on successful healthcare service outcome [13, 14]. According to the World Health Organization (WHO), the referral form is designed to facilitate communication and should clearly record patient identification, clinical findings, treatment given, specific reason for referral and clearly mention the initiating and receiving facilities. Also: where it is possible to book an appointment or accompany the patient in case he/she is in a critical condition. Every facility should keep a referral register to monitor referrals. The same information is required for counter referral in addition to the description of follow up care [15]. Faramarzi [10] concludes his research with: “A “qualified referral letter” should include the description of chief complaint, description of associated symptoms, relevant physical findings, past medical history, drug history, family history, and reasons for referral”. While the research conducted in South-East England by Smith and Xiang assessed the quality of referral letters using the following quality criteria: presence of “blood pressure, body mass index, past medical history, medication history, provision of clinical information and clarity of reason for referral” [11]. Health level seven (HL7) is a standard defining electronic messages that allows healthcare information exchange, interoperability to improve care delivery and optimization of clinical and administrative data. Patient referral messages should include patient demographic, clinical information, treatment, patient referral message or reason for referral, clinical history, and referral priority status [16].

Referrals in developing countries are still a burden because they hardly follow any process or procedure at the primary care level which emphasizes the need for improvement that will lead to better quality of care [17, 18]. A referral letter is the sole information available regarding the patient on arrival and considerable frustration has been expressed by specialists because they are deprived from relevant quality information content [19]. Furthermore, there are serious concerns about whether and when to refer a patient for specialized care due to inadequate primary care investigation, which leads to unnecessary and premature referrals and results in ambiguous expectations or adverse outcomes and fragmented care. A referral guideline accompanied by a decision support process would improve this [20, 21]. Moreover, highly prevalent gaps in coordination and unsatisfactory communication between primary care and specialized care lead to care delays, poor access to specialized care, duplicating of tests, unnecessary costs, inefficiencies and sub-optimal patient outcomes [22–24]. In Rwanda, the officially approved structured referral letters don’t have mandatory and optional fields, which may lead to the healthcare provider filling in some less critical information and omitting other fields which are crucial for the continuity of care. The outcome of this research will be a clear understanding of formats and completeness of referral letters, hence, will contribute to the existing literature especially in patient management and healthcare information exchange to the benefit of the Ministry of Health, hospitals, healthcare providers, patients, and researchers. *This study aims to enhance the provision of quality healthcare services through an assessment of the existing paper-based referral letter formats and completeness to improve health information exchange, coordination, and continuity of care based on data collection from the hospitals in the urban environment of Kigali.*

## Methods

### Setting

Our study was conducted in eleven health facilities in Kigali city. Eight public hospitals (two referral hospitals (RH1 and RH2), three district hospitals (DH1, DH2, and DH3), three health centers (HC1, HC2, and HC3) and three private hospitals (PH1, PH2, and PH3). The settings were purposely selected to include public and private health facilities in an urban setting that provide primary, secondary and tertiary healthcare located in the same catchment area. In October 2019, the Rwanda Ministry of Health (MoH) approved and published seven public structured referral letter formats namely: (i) external transfer form, (ii) neonatal transfer form, (iii) antenatal care (ANC), delivery and postnatal care (PNC) external transfer form, (iv) internal transfer form, (v) patient monitoring transfer form, (vi) health center transfer form to community health worker, and (vii) community health worker transfer form to health center. On the other hand, every private hospital designed its own referral letter.

### Study design

A retrospective exploratory research was conducted to evaluate the completeness of referral letters in the selected hospitals from May 2021 to October 2021. A purposive sampling method was used to select referral letters from the records, whereby only patient files containing referral letters were considered. The instruments that were used are data capture sheets specifically designed according to the contents or variables of the referral letters whereby every single variable that was completed was assigned a YES and missing variables were assigned a NO.

### Data collection and procedure

After obtaining ethical clearance from the Ministry of Health, the researcher visited the selected hospitals and had a meeting with their representatives. Further, she explained the purpose of the study and after obtaining permission from all hospitals, a designated assistant was appointed. The designated assistant helped the researcher to find the referral letters.

Referral formats were assessed at first, all available referral letters were retrieved, and the contents were compared with the WHO standard referral to assess whether all important variables were present. Hence, we have examined ten different types of referral letters. (i) Referral/counter referral form “*Fiche de référence / Contre référence*” is the old version designed in French which is still predominantly used in public health facilities. Five updated structured referral letters: (ii) external transfer form, (iii) internal transfer form, (iv) ANC, delivery, and PNC external transfer form, (v) neonatal transfer form, and (vi) patient monitoring transfer form. (vii) DH2 is a self-designed referral letter used in a district hospital. (viii) PH1, (ix) PH2, and (x) PH3 are also self-designed referral letters used by the three private hospitals selected.

Specific data capture sheets were designed according to the referral letter templates found at each hospital; they were then used to capture the completeness of the referral letters. The two referral hospitals have an archive room where you can find all patients’ files. Whereas the district hospitals, health centers as well as private hospitals store all hospital documents including patients’ files, management and financial records together in boxes in one room. Sometimes a month and year was written on the box. Recent patients’ files were found in different areas including outpatient department, in patient (hospitalization) wards, emergency and finance departments. The researcher reviewed all patients’ files from July 2019 to July 2021, only patients’ files containing a referral letter were considered and patients files without a referral letter were excluded. Some health facilities keep printed booklets and when they transfer a patient, a copy of the transfer letter would stay in the booklet. However, once the booklets are out of stock, which was mostly the case during Covid-19 pandemic, a photocopy of the form was given to the patient and no copies were kept at the referring facility. Nevertheless, one private hospital prints referral letter templates and hands them over to the patient while no soft or hard copy is kept at the referring facility, and no referral feedback was received at a later stage.

### Sample size

The estimation of the sample size was challenging due to poor record keeping and difficulties in retrieving referral letters. The sample size was drawn from an unknown population. To calculate the sample size needed, Cochran (1963:75) equation was used n_0_ = Z^2^ pq / e^2^ where: Z is the confidence level estimated at 95% = 1.96, p is the estimated proportion of an attribute that is present in the population *p* = 0.5, q = 1-p, and e is the margin error 0.03 were considered for sufficient statistical relevance. Hence, n_0_= (1.96)^2^ (0.5) (0.5) / (0.03)^2^ = 1067.

### Analysis

Microsoft Excel (365 Version) was used to capture the data and descriptive analyses were conducted by counting the number of YES responses and calculating the related average percentages.

## Results

### Referral letters formats

During the assessment of formats, ten different structured referral letters formats were found. Contents or variables were grouped into elements (e.g., Patient identification includes: Patient name, date of birth, gender, address, telephone number, district, sector, cell, and village, serial number in register or electronic medical record identification number), then these elements were further compared. The external transfer form (R1) is used in outpatient departments (OPD) and for inpatients to be referred to another health facility. Neonatal transfer forms (R2) are used to refer newborns. ANC, delivery, and PNC external transfer forms (R3) are used in obstetrics and gynecology departments to refer pregnant women having complex prenatal obstetric conditions, complicated delivery and postpartum problems to another health facility. The internal transfer form (R4) is used to refer patients inside the same health setting. Finally, the patient monitoring transfer form (R5) is used to monitor patients during transportation. The other two formats referring to and from the community health workers were excluded from this review.

Table 1 shows a comparison of the structured referral letter content of the World Health Organization (WHO), Health level seven (HL7), five updated structured referral letters (R1 to R5), the old format Referral / Counter referral form (R6), Self-designed referral letters for a district hospital (DH2) and three private hospitals PH1, PH2 and PH3.

**Table 1:** Referral letters standards comparison

Elements	WHO	HL7	R1	R2	R3	R4	R5	R6	DH2	PH1	PH2	PH3
Patient Identification	√	√	√	√	√	√	√	√	√	√	√	√
Initiating facility	√	√	√	√	√	√	√	√	√	√	√	√
Referred to facility	√	√	√	√	√		√	√	√	√	√	√
Clinical history	√	√	√	√	√			√	√			
Findings	√	√	√	√	√	√		√	√	√	√	√
Treatment given	√	√	√	√	√	√	√	√	√	√	√	√
Reason for referral	√	√	√	√	√	√		√	√	√	√	√
Documents accompanying referral	√	√	√	√	√				√			
Referral priority		√	√	√	√							
Maternal history				√	√							
Labor details				√	√							
**Back referral/counter referral**												
Initiating facility	√	√	√	√	√			√			√	
Referred to facility	√	√	√	√	√			√			√	
Patient Identification	√	√	√	√	√			√			√	
Patient history	√	√	√	√	√			√				
Special investigations and findings	√	√	√	√	√			√				
Diagnosis	√	√	√	√	√			√				
Treatment / operation	√	√	√	√	√			√				
Medication prescribed	√	√	√	√	√			√				
Please continue with: (Meds, Medical prescription, follow-up, care)	√	√	√	√	√			√				

Considering the WHO format as the global standard reference, the above comparison shows that all relevant information needed throughout the patient referral cycle has been included in the updated Rwandan structured referral letters. If the information content of the WHO referral standard is considered to be (100%), R1 has 112.5% due to the addition of “referral priority”. More information has been added to R2 and R3 both leading to 137.5% to support newborns and women throughout pregnancy, delivery and post-partum referrals. R4 holds limited referral information 62.5% as the patient is referred inside the same facility. Hence, the patients’ record can be obtained by the receiving department because it is in the same health setting. Finally, R5 also holds 50%, as it is limited to the information needed throughout the transportation journey of patients in ambulances. R1, R2, and R3 back referral information contents are the same as the WHO standard. With regards to the old format (R6), it has 87.5% of referral’s content in comparison to WHO while the back referral holds the same. Finally, DH2, PH1, PH2, and PH3 have 75%.

### Study sample

The study population included 2,304 referral letters (Table 2). Eight different referral letter formats were found to be in use within the eleven health facilities selected. The private hospital PH3 did not keep a copy soft or hard of the outgoing referral letters sent while the original document was given to the patient.

**Table 2:** Summary of study sample

SN	Referral letters format / Hospitals	RH1	RH2	DH1	DH2	DH3	HC1	HC2	HC3	PH1	PH2	PH3	Total
1	External RL	8	89	7			67	72	64				**307**
2	Internal RL	48	21	10									**79**
3	ANC, Delivery, PNC RL	1	9				61		66				**137**
4	Referral/counter RL	202	90	160		242	32		114				**840**
5	DH2 RL				149								**149**
6	PH1 RL									575			**575**
7	PH2 RL										217		**217**
8	PH3 RL											0	**0**
**Total**		**259**	**209**	**177**	**149**	**242**	**160**	**72**	**244**	**575**	**217**	**0**	**2304**

*RH* Reference Hospital, *DH* District Hospital, *HC* Health Center, *PH* Private Hospital, *RL* Referral Letter

### Completeness level of referral letters

The average completeness of referral letters was found to be: Referral / Counter referral forms at 46.0%; external transfer forms at 58.8%; antenatal, delivery, and postnatal external transfer forms at 47.5%; internal transfer forms at 46.6%; district hospital (DH2) at 73.4%; while private hospitals (PH1, PH2 and PH3) referrals were completed at (97.7%); (70.7%); and (0.0%) respectively. A major deficit was observed in the completeness of counter referral information in all the hospitals. Further analyses found a negative correlation between the number of variables on the referral forms and the level of completeness though not statistically significant (*r* = -0.543, *p* = 0.208).

### Referral / Counter referral form completeness (Table 3)

Referral / Counter referral form “*Fiche de référence / Contre référence*” is an old format designed in French that was still predominantly used in public health facilities in the year 2021 despite the introduction of the updated structured referral letters in October 2019. Our findings shows that the Referral / Counter referral form holding twenty-two variables was completed on average at (46.0%), with (83.3%) of referring information and only (3.9%) of counter referral information (Table 3) (Additional file 3).

**Table 3:** Referral / Counter referral form

Referral / Counter referral (*n* = 840)	Referral Hospitals		District Hospitals		Health Centers		Average	Total Average
	**RH1*n*** **= 202**	**RH2*n*** **= 90**	**DH1*n*** **= 160**	**DH3*n*** **= 240**	**HC3*n*** **= 114**	**HC1*n*** **= 32**		
Patient Identification (%)	64.8	59.0	56.5	63.5	87.7	88.5	66.2	
Clinical Information (%)	76.9	73.6	88.4	71.0	77.9	65.6	76.7	
Referring Healthcare provider details (%)	100.0	100.0	100.0	100.0	100.0	100.0	100.0	
Counter referral (%)	6.5	0.8	2.5	0.0	0.0	47.3	3.9	
Average completeness of referring (%)								**83.3**
Average completeness of counter referral (%)								**3.9**
**Total average completeness (%)**								**46.0**

*RH* Reference Hospital, *DH* District Hospital, *HC* Health center, *n* Count

### External referral letter completeness (Table 4)

The external referral letter has sixty-one variables and was completed on average at (58.8%) with (62.0%) of the referring information while counter referral was completed on average at (42.6%). (Additional file 1) Some major loopholes were observed in the completeness of transfer details whereby the calling time of the staff at the receiving facility was completed at (6.5%), the name of the staff contacted at the receiving facility (6.2%). In addition, the phone numbers of the receiving facility were almost not recorded at all (5.5%). Furthermore, the transfer emergency which records the time an ambulance was called was completed at (3.3%) and the time of ambulance departure from the referring facility at (2.6%). The most vital information on the referral letters is clinical information which was completed at (44.3%). Finally, the information about the vital signs was the most poorly completed at (32.7%) (Table 4).

**Table 4:** External referral form

External Transfer Form (*n* = 307)	RH1 *n* = 8	RH2 *n* = 89	DH1 *n* = 7	HC3 *n* = 64	HC1 *n* = 67	HC2 *n* = 72	Average
Patient Identification (%)	71.4	90	100	99.8	98.7	86.7	92.9
Transfer details (%)	54.9	54.7	68.3	57.3	62.3	46.8	55.3
Clinical Information (%)	67.9	43	53.1	40.8	37.5	51.8	44.3
Vital signs (%)	29.7	30.2	41.1	48.8	32.1	21.5	32.7
Referring healthcare provider details (%)	97.5	92.1	91.4	95.9	83.9	64.4	84.8
Referral feedback & Counter referral (%)	24.2	22.8	0	48.9	43.5	66.8	42.6
Average completeness of referring (%)							**62**
Average completeness of counter referral (%)							**42.6**
**Total average completeness (%)**							**58.8**

*RH* Reference Hospital, *DH* District Hospital, *HC* Health center, *n* Count

### ANC, delivery, and PNC external referral form completeness (Table 5)

The ANC, delivery, and PNC external transfer letters that hold 103 variables were completed on average at (47.5%) with (46.7%) of referring information and (53.9%) of counter referral data. The information on the treatment given to the patient from the referring health facility (1.0%) was the least completed followed by the results of the investigations done (4.1%) and vaginal examination (17.2%) (Table 5) (Additional file 2).

**Table 5:** ANC, Delivery, and PNC external referral form

ANC, Delivery, and PNC external transfer form (*N* = 137)	Referral Hospitals		Health Centers		Average
	**RH1*N*** **= 1**	**RH2*N*** **= 9**	**HC3*N*** **= 66**	**HC1***N* **= 61**	
Patient identification (%)	77.8	66.7	71.4	68.9	**70.0**
Clinical information (%)	64.3	39.7	46.8	45.7	**45.9**
Obstetric history (%)	50.0	59.4	65.0	54.9	**60.0**
Maternal vital signs (%)	85.7	57.1	52.6	55.3	**54.3**
Abdominal examination (%)	100.0	86.1	78.0	79.9	**79.6**
Vaginal examination (%)	0.0	33.3	16.8	15.5	**17.2**
Investigation results (%)	25.0	8.3	0.9	6.6	**4.1**
Received treatment at health facility (%)	14.3	3.2	0.0	1.6	**1.0**
Referring care provider (%)	100.0	88.9	93.2	83.1	**88.4**
Referral feedback/Counter referral (%)	0.0	54.9	55.4	44.7	**53.9**
Average completeness of referring (%)					**46.7**
Average completeness of counter referral (%)					**53.9**
**Total average completeness (%)**					**47.5**

*RH* Reference Hospital, *HC* Health Center, *n* Count

### Internal referral form completeness (Table 6)

The internal transfer letter composed of 32 variables was completed on average at (46.6%). The referral hospital RH1 and district hospital DH1 have never recorded the receiving healthcare provider details while clinical information was completed on average at (56.3%) (Table 6) (Additional file 4).

**Table 6:** Internal referral form

Internal Transfer Form (*n* = 79)	RH1 *n* = 48	RH2 *n* = 21	DH1 *n* = 10	Average
Client Identification (%)	39.2	31.0	30.0	35.8
Transfer Details (%)	65.6	22.2	50.0	52.1
Clinical Information (%)	60.4	42.9	65.0	56.3
Referring Healthcare Provider (%)	91.7	27.6	80.0	73.2
Receiving Healthcare Provider (%)	0.0	59.0	0.0	15.7
**Total Average**				**46.6**

*RH* Reference Hospital, *DH* District Hospital, *n* Count

### Self-designed referral form completeness (Tables 7, 8 and 9)

One of the public district hospitals DH2 (Table 7) (Additional file 7) was found with a self-designed referral letter with 13 items. It was completed on average at (73.4%) with referring information completed on average at (91.8%) while the information about counter referral was not at all completed (0.0%). The referral letters of the private hospitals PH1 (Table 8) (Additional file 6) and PH2 (Table 9) (Additional file 5) (designed in French) both having eleven variables were completed on average at (97.7%) and (70.7%) respectively. PH1 has no counter referral on their designed referral letter whereas PH2 completed on average the referring information at (94.3%) with no referral feedback at all (0.0%). The private hospital PH3 has no record of referrals in their archives (Tables 7, 8 and 9).

**Table 7:** The district hospital DH2 referral form

DH2	Average	
Patient Identification (%)	99.8	**91.8**
Transfer Details (%)	87.2	
Clinical Information (%)	81.5	
Referring Health care Provider (%)	98.7	
Counter referral (%)	0	**0**
**Total average (%)**		**73.4**

**Table 8:** Private hospital PH1 referral form

PH1	Average
Patient Identification (%)	99.8
Clinical Information (%)	93.6
Referring Healthcare Provider (%)	99.7
**Total average (%)**	**97.7**

**Table 9:** Private hospital PH2 referral form

PH2	Average	
Patient Identification (%)	88.6	**94.3**
Clinical Information (%)	94.3	
Referring Healthcare Provider (%)	100	
Counter reference (%)	0	**0**
**Total average (%)**		**70.7**

## Discussion

The present analysis was the first Rwandan assessment of the referral letter formats and completeness from a broad range of specialties in both public and private sectors. This study investigated the formats and completeness of paper-based referral letters in eleven health facilities in Kigali city.

### Formats of referral letters

Eight different referral letter formats were found to be in use within the selected health facilities. No uniformity was observed in the design of the referral letters making it harder to the receiving party to immediately grasp key information. Specialists have expressed their frustration towards the formats of the referral letters and emphasized that a good format would facilitate quick and easy retrieval of comprehensive information [17, 25]. It was therefore suggested that the use of structured referral letter formats may facilitate an efficient communication between healthcare providers [26]. To overcome these challenges, the Rwanda Ministry of Health updated the structured referral letters templates in 2019. Despite the update, the old format Referral form / Counter referral was still predominantly used in public hospitals in 2021. The investigator observed that some hospitals had in their stock old format (Referral / Counter referral form) printed booklets which may be the reason to its popular use, the same was noticed at DH2. Another motive might be poor dissemination strategies and lack of training.

Further observations were made on the ANC, delivery, and PNC external transfer and neonatal formats for their font size being too small with insufficient space for handwriting and too many variables, which may have led to their rare usage. Instead, practitioners preferred to utilize the external transfer form as a replacement for the former. This is in line with the observation of A. Janati et *al.* stating that referral letters directed to gynecologists are more specific, however, they do not provide enough space to support criteria for referral writing styles [27]. It was further observed that one of the public district hospitals DH2 has ever since used its own designed referral letter format despite several updates of the national structured referral templates. The format designed specifically to record patient information during transportation in an ambulance were not found in use in any of the public hospitals. Only patients with emergency cases get the ambulance transport facilitation explaining the importance of the patient transfer monitoring form to record information during transportation for the continuity of care. Ezhumalai et *al.* emphasized that referral as a process and care continuum of the patient during transportation is vital to a good outcome [28]. It is therefore paramount to include the information recorded during ambulance transportation in the patient’s file for further reference.

### Referral completeness

All the items inserted in the structured referral letters are important and need to be filled to allow smooth information transfer and continuity of care. Unfortunately, absence of some information at the receiving end leads to fragmented care and duplication of examination procedures [20, 21]. This may sometimes compromise patient safety, outcome and it leads to unnecessary loss of time and resources [22–24]. The findings of this study showed that the average overall completeness of the referral letters varies between 46.0% and 97.7% with an average of 71.8%. These results are in agreement with the findings of the study conducted in Hamilton that reported a completeness of 75.6% of the hospital referral files [29]. Much lower results were found in other studies. A study conducted in the Royal Hobart hospital – Australia reported that 58.8% of the information was filled in on the referral notes [30]. Similar findings were also obtained in a study done by Usher et al. which found 58.3% completeness of the files [31]. It might therefore generally be determined that referral letters are inadequately completed. However, it is not known whether the reason for these omissions is due to oversight or lack of time of the referring provider who might sometimes decide not to fill some data on the referral files, inappropriate formatting of the referral forms, or patient related factors such as missing information. It is therefore important to explore the reasons for those oversights found in the referral letters.

It is essential for healthcare providers to complete all the variables of referral letters for a smooth continuity of care. Only PH1 had few referral letters completed with all variables and the other six referral letter formats had at (least) one or more variables missing. This findings concur with the results of Kipkulei and Lotodo (2019) conducted in Kenya whereby only 1% of the referral letters contained all the required information [32]. It was further observed that referral letters missed one or more variables with the exception of patient name which had been written in all referral letters, this is in agreement with findings of previous studies [27, 32, 33].

Also, although users were familiar with the old structured referral letter “Referral / Counter referral form” as shown in the results (Table 3) (Additional file 3) the file number “*numéro du dossier*” was not completed even when filled-in, it was still confusing because some filled in the patient identification number at the referring hospital while others filled in the number of the page in the pre-printed booklet. This situation might have been caused by the fact that there was no unique patient identifier as well as referral register of incoming and outgoing referrals in all the health facilities.

In 2005, Rwanda established local administration entities structured in four tiers (District, sector, cell, and village) [34]. The patient’s address should comply with this structure, which all the citizens are not yet acquainted with, hence this might be one of the reasons for the reluctancy in completing the addresses. It was further observed that the “*traitement reçu*” which means “received treatment” was not completed, the reason might be because all the patients that visit primary care in acute condition are immediately referred without any treatment given at the primary care. It might be a good decision in emergency cases for the patient to be attended by a specialist at a higher level to avoid care delay, however, it might also be a premature referral without thorough primary investigation. Some guidelines for primary care pre-referral investigation would clarify this situation. Healthcare providers did not miss to fill in the date on the Referral / Counter referral form compared to 91% from a study conducted in Tunis El Manar university hospital whereas the patient’s medical history fields were completed in 48.5% of the cases in our study compared to 47% in a previous study [35].

The new template called *external transfer form* is used by all the departments except neonatology and gynecology. This updated template holds more information than the old one, some transfer details are included to smoothen the referral process, they are: calling time at the receiving facility, name of the staff contacted at receiving facility, phone number of the staff contacted at receiving facility, time ambulance called, time of departure from referring facility. All these details were completed in less than 7% of the samples, a reason might be the lack of triage which is the process of sorting and allocating patients according to their needs thus complementing the coordination of care. Vital signs were included as well on the new templates, however, some parameters such as person with disability - record containing the type of disability, height, mid-upper arm circumference (MUAC) were completed at less than 6%. A possible reason for this low completeness could be that the description of the type of disabilities is missing in the referral guidelines. Since this format is used for adults, this might explain the reason why the height and MUAC were not filled in. Comparing the external transfer form results with a similar study conducted at a teaching hospital in Lagos, Nigeria shows that weight was completed at 32.9% vs. 18% in Lagos, vital signs 32.7% vs. 14%, physical examination findings 61.6% vs. 44%, diagnosis 95.4% vs. 88%, height 5.9% vs. 0.0%; treatment given 35.5% vs. 92%, laboratory 11.4% vs. 10% [36].

The ANC, delivery, and PNC transfer forms were designed specifically for obstetrics and gynecology referrals, these new structured referral templates are well detailed. However, it might be hard to criticize their corresponding completeness since they are used to refer pregnant women in three phases (antenatal, delivery, postnatal), therefore it would be possible that some information might not be needed in one case or another. Hence, clear guidelines would be necessary for patient safety and auditing purposes. Some transfer details required to be filled in for the three phases were completed in less than 7% of the cases including: name of next of kin, telephone of the next of kin, calling time at receiving facility, name of the staff contacted at receiving facility, phone of the staff contacted at receiving facility, if emergency - time ambulance called, time of departure, copy of partograph attached, if person with disability - the type of disability. These details are important to improve communication between providers and to assure coordination of care.

It was further found that the average completeness of the “ANC, delivery, and PNC transfer” forms collected in the present study was higher than the obstetrics and gynecology format assessed in a rural hospital in Iran [27]. Specifically, patient identification numbers were completed at 22.6% compared 0.0% in the latter study; patient age was 100% versus 44.7%, reason for referral 100% versus 71%, telephone number of the referring provider 62.0% versus 0.0%, and name of the receiving provider 5.8% versus 0.0%. The authors highlight that the above details are important for inter-provider communication.

The internal transfer form had some information fields on patient identification which were not completed, the reason might be that these information are already in the patient record, hence providers skip them to avoid useless repetition. However, some very important information for continuity of care was completed at less than 20% including the name and phone number of the staff contacted at the receiving service. In the self-designed referral letters DH2, PH1 and PH2, referral information was completed over 90% with the exception of referral feedback information.

Referral feedback or counter referrals completeness was 0.0% for DH2, PH1 because there was no slot for recording the specific information on counter referral. PH2 scored 0% and Referral / Counter referral form 3.9%. Similar results were obtained in the study that was conducted in Sri Lanka showing that only 7.5% of the referral feedback was received [37]. Contrary, an improvement was observed regarding referral feedback in the updated formats with 53.9% for ANC, delivery and PNC external transfer letter, and 42.6% for the external transfer form. This could be explained by the introduction of the referral insurance reimbursement scheme which is accepted only when the referral feedback is available. Nevertheless, the counter referral forms were kept at the finance department for compensation and were never added to the patient medical records. Moreover, when the same patient revisits the primary care entity, he is treated as a new patient. This constitutes a major handicap for the coordination and continuity of care.

It was further observed that the following updated referral letters: ANC, delivery, and PNC external transfer form, external transfer form, and Internal transfer contain 103, 61, and 32 variables respectively. Hence, their corresponding completeness was below 60% while the self-designed referral letters DH2, PH1, and PH2 with 13, 11 and 11 variables respectively, were the only ones with completeness above 70%. This might be explained by the fact that the new forms having more variables would take too long to fill in compared to the self-designed referral letters. Therefore, many health professionals would consider filling them entirely to be too time consuming. They would prefer to fill information that they deem necessary while leaving other sections uncompleted.

### Limitations

This assessment mainly focused on data completeness while other aspects such as data quality, including accuracy and reliability, were not evaluated. Due to inadequate hospital record keeping, we were unable to follow specific patients throughout their complete referral journey to check whether the referral completeness was upgraded from primary care to secondary care. Another limitation was the possibility to assess the reasons behind the incompleteness and whether the health care professionals were satisfied about the updated structured referral formats. We were also unable to determine the exact reasons why many variables on the referral letters were not filled in, if it was due to the workload and the time it takes to fill in all the variables, but this should be confirmed by further research.

### Recommendations

The following recommendations were proposed:

- Dissemination of structured referral letters in hospitals.
- Introduction of incoming and outgoing referral register in each care institution.
- Elaboration of national referral guidelines which include but are not limited to the use of a referral decision support system, a triage system to facilitate the coordination between the facilities, including detailed steps for pre- and counter referral investigations; a referral rejection process in case the referral letter misses key information; a referral feedback facilitation; methodology for referral letters archiving in the patients’ records.
- Including the referral system in the education modules for medical and para-medical professions and organizing regular training sessions on the referral system in continuing education programs will improve the completeness.

## Conclusion

The study found that there were inconsistencies in the use of the available *referral formats* by hospitals. It also showed and quantified the deficits in the completeness of referral letters. Few variables were completely filled in on the referral letters, whereas others were poorly or not completed at all. Emphasis on referral completeness and referral feedback may improve patient outcome. The study does not include an investigation on organizational issues but concentrates on all aspects directly related to referral formats and their completeness. An assessment of perception of healthcare providers on the referral system and the implementation of an electronic referral system are subjects of our further research.

## Supplementary Information

**Additional file 1.** External referral form.

**Additional file 2.** Antenatal, Delivery, and Postnatal external transfer form.

**Additional file 3.** Referral / Counter referral form.

**Additional file 4.** Internal referral form.

**Additional file 5.** Private Hospital PH2.

**Additional file 6.** Private Hospital PH1.

**Additional file 7.** District Hospital DH2.

## Data Availability

All data generated or analyzed during this study are included in this published article.

## Abbreviations

ANC: Antenatal Care
CBHI: Community-based Health Insurance
CHW: Community Health Worker
DH: District Hospital
HC: Health Center
HL7: Health Level Seven
MUAC: Mid-upper Arm Circumference
OPD: Outpatient Department
PH: Private Hospitals
PNC: Postnatal Care
RH: Referral Hospital
RL: Referral Letter
WHO: World Health Organization
